# Urgent need for substance use disorder research among understudied populations: examining the Asian-American experience

**DOI:** 10.1093/haschl/qxad058

**Published:** 2023-10-25

**Authors:** Sugy Choi, Sahnah Lim, Simona C Kwon, Chau Trinh-Shevrin, Charles J Neighbors, Stella S Yi

**Affiliations:** Department of Population Health, NewYork University Grossman School of Medicine, New York, NY 10016, United States; Department of Population Health, NewYork University Grossman School of Medicine, New York, NY 10016, United States; Department of Population Health, NewYork University Grossman School of Medicine, New York, NY 10016, United States; Department of Population Health, NewYork University Grossman School of Medicine, New York, NY 10016, United States; Department of Population Health, NewYork University Grossman School of Medicine, New York, NY 10016, United States; Department of Population Health, NewYork University Grossman School of Medicine, New York, NY 10016, United States

**Keywords:** addiction, Asian American, ethnic groups, disparities

## Abstract

Substance use disorder (SUD) among Asian Americans is understudied. Our review of National Institutes of Health–funded projects reveals a striking underrepresentation of research focused on SUD in this population, possibly perpetuated by the pervasive societal myth that Asian Americans are a healthy community. Moreover, the limited availability and disaggregation of data on SUD among Asian Americans further hinder our understanding of prevalence rates, treatment utilization, and associated disparities—thereby limiting opportunities for prevention and intervention. In light of these findings, our review serves as a crucial call to action, emphasizing the urgent need for increased research efforts and resources to address the significant gaps in knowledge and inform effective interventions for addressing SUD among Asian Americans.

## Introduction

Substance use disorder (SUD) is a pressing medical condition characterized by the harmful consumption of substances like drugs or alcohol, encompassing a wide range of substances, including opioids, cocaine, methamphetamine, and cannabis. In the United States, SUD has emerged as a significant public health concern, particularly due to the opioid epidemic. However, within this nationwide focus, it is essential not to overlook certain populations that have been both understudied and underserved in the context of SUD.

Research aimed at understanding the experiences of underserved populations plays a pivotal role identifying barriers to access and developing targeted strategies to enhance health care delivery and reduce disparities related to SUD. Recognizing this, we focus our research on examining the unique challenges faced by the Asian-American population in relation to SUD.^1,2^ By doing so, we envision that the broader insights gleaned from our study can be applied meaningfully to other understudied groups confronting similar hurdles, such as issues related to data disaggregation, language barriers, and harmful stereotypes.^3^

## Examining the Asian-American experience

According to the 2020 National Survey on Drug Use and Health (NSDUH), approximately 1.5 million Asian adults aged 18 years or older had an SUD.^4^ Cannabis emerged as the most frequently used illicit drug in the past year among this population.^5^ In 2020, there was a significant increase in the incidence rate of past-month alcohol and cocaine use and tranquilizer misuse among Asian-American individuals compared with White individuals in 2016.^6^ Both adult and youth Asian Americans face unique challenges and disparities in accessing appropriate support and treatment for SUD.^7^ Asian Americans, the fastest-growing racial-ethnic group in the United States,^8^ often encounter challenges in accessing formal support for substance use treatment. These challenges can be attributed to a cultural emphasis on self-reliance and a strong desire to maintain positive images and values regarding SUD and mental health.^9-11^ Additionally, a significant percentage of Asian-American adults are foreign-born (71%),^8^ and approximately one-third of Asian-American adults have limited English proficiency (LEP),^12^ further complicating their access to appropriate substance use treatment services and interventions. Structural and system-level factors, including the lack of a bilingual and bicultural workforce, resources, and programs specifically tailored to meet the needs of Asian-American communities, play a significant role in perpetuating disparities in substance use treatment access. The underinvestment in culturally and linguistically sensitive health services, campaigns, and outreach efforts further marginalizes Asian Americans and contributes to their reluctance to seek formal support for substance use treatment and mental health. Consequently, some individuals may turn to substance use as a coping mechanism for their emotional distress.^13^

Recent trends in overdose mortality among Asian Americans show a concerning pattern, with overdose deaths increasing by 967% from 2007 to 2019 due to the concomitant use of opioids and cocaine.^14^ Additionally, the prevalence of methamphetamine use without injection doubled from 2015 to 2019 among Asian Americans, along with several other racial and ethnic minority groups, highlighting an upward trend in substance use.^15^ On the other hand, there was a disparity in the patterns of substance use and misuse between Asian and White Americans during the COVID-19 public health emergency in 2020, with a marked increase in the incidence rate among Asian Americans.^6^ While these increases may be lower compared with other racial groups, it remains concerning that Asian Americans exhibit the lowest rates of substance use treatment utilization for SUD among all racial and ethnic groups.^16^ The surge in anti-Asian sentiment and experiences of discrimination, exacerbated by the COVID-19 public health emergency, have contributed to a decline in mental health among Asian Americans, with approximately 30% of Asian Americans reporting having experienced discrimination.^17^ This deterioration in mental well-being may, in turn, increases the susceptibility of Asian Americans to substance use as a coping mechanism.^18-22^ This chain of effects highlights the complex interplay between societal factors, mental health, and substance use in the community.

Asian Americans in need of SUD treatment underutilize SUD services. They are less likely to recognize that they have an SUD or seek behavioral health services.^23^ Asian-American populations with SUD who do enter treatment receive significantly less psychosocial support compared with their White counterparts and have lower treatment retention rates than other racial/ethnic groups. This is, in part, due to the treatment or services not being culturally tailored, resulting from a lack of evidence-based treatment and strategies specifically designed for Asian-American populations.^9^ Limited availability of treatment options in certain neighborhoods further exacerbates these disparities.^9^ It is important to note that completion rates vary among Asian Americans based on factors such as state population, density, and social networks.^10^ Methamphetamine and opiate users, particularly among Asian Americans, exhibit lower completion rates than alcohol users.^24^ The lack of data disaggregated by Asian ethnic groups has impeded understanding of the nature and prevalence of SUD within Asian-American populations. Based on prior literature revealing social and health disparities by Asian ethnic group (eg, Chinese, Korean, Taiwanese, Pakistani, Vietnamese, Cambodian), we assert that SUD treatment and completion rates differ by subgroup as well.

## Examination of funding landscape of Asian-American SUD research

We highlight the urgent need for SUD research among Asian Americans and provide recommendations for addressing this issue (Table 1). First, we examined the funding levels for research on Asian Americans and SUD by the National Institutes of Health (NIH). The NIH is widely recognized as the largest funding source for biomedical and health research in the United States.^25^ The NIH has emphasized the importance of advancing health equity. It recognizes the need to address health disparities and promote inclusivity in research to ensure that all population groups have equal access to health care advancements and benefit from scientific discoveries. The NIH–National Institute on Minority Health and Health Disparities (-NIMHD) specifically focuses on reducing health disparities and promoting health equity among racial and ethnic minority populations. One of the priority areas of NIMHD is to standardize and disaggregate subpopulation data. The importance of disaggregating data by specific ethnic and racial groups, such as Asian ethnic groups, has gained attention as it allows for a more accurate understanding of the diverse experiences, health outcomes, and health care needs within these populations.^3,26^ This recognition stems from the realization that aggregating data at a higher level can mask important disparities and hinder the development of targeted interventions and policies.^4^ Examining NIH-funded research is crucial not only because it advances public health and improves the well-being of individuals and communities but because it can provide training and education for the next generation of scientists and health care professionals who specialize in Asian-American health. A prior study shows that the overall number and amount of research grants focusing on Asian Americans, Native Hawaiians, and Pacific Islanders have increased from 1992 to 2018, although they represent only 0.17% of the NIH budget.^2^ Yet, these communities represent 6% of the US population and are projected to double in size by 2060.^27^

**Table 1.** Key recommendations and action steps for advancing multifaceted approach to address substance use disorder in Asian American communities.

1. Increase National Institutes of Health funding and support for research on Asian-American communities and substance use disorder (SUD), especially for interventions targeting overdose prevention among specific Asian communities as opposed to a general focus on “Asian Americans.”
2. Improve data collection for limited-English-proficiency Asian-American individuals and disaggregate data for different Asian-American ethnic groups. Report consistent data to gain a comprehensive understanding of SUD prevalence and treatment needs among these subgroups. Additionally, support the expansion of data collection in multiple languages.
3. Promote intersectional approaches by examining trajectories across the lifespan at the ethnic group level. This approach will enhance the development of culturally tailored interventions that consider cultural norms, immigration status, language needs, ethnic background, age, and the availability of mental health support within the family and community.
4. Strengthen community-based and patient-centered approaches, fostering synergy between research and practice. This collaboration should facilitate the timely translation of research findings into effective interventions and policies that directly benefit Asian-American communities. Utilize the existing relationships that community-based organizations have to reach limited-English-proficiency Asian-American individuals, taking into account mistrust and fear of public shame.
5. Develop and test tailored interventions designed to prevent substance use and reduce the harms associated with substance use among Asian-American subgroups. These interventions should take into consideration cultural norms, language, ethnic background, age, and other relevant factors to effectively address the unique needs of each subgroup.

Recognizing the need for more equitable research practices, the recommendations can be revised to encompass all understudied populations and underscore the importance of considering ethnic subgroups and cultural nuances within each community, aiming to address the disparities and challenges surrounding SUD comprehensively.

Through a systematic search of NIH grants, we enumerated funding support for Asian Americans and SUD from January 1985 to September 2023. Our analysis focused on identifying administering institutes and centers, studied substances, Asian ethnic groups, special populations, research designs, and themes. A total of 534 records on NIH RePORTER were retrieved using the search term “Asian Americans” and/or “substance use disorder”; “Asian Americans” and/or “alcohol”; “Asian Americans” and/or “opioid”; “Asian Americans” and/or “illicit”. During the screening process, we collapsed the same projects across multiple years into 1 record and excluded in vivo studies. Ultimately, we included 30 unique records in our analysis after removing abstracts that did not meet our criteria. After the full abstract screening, we grouped records into 2 categories: (1) studies focused solely on Asian Americans (*n* = 17) and (2) studies focused on both Asian Americans and non–Asian Americans, including Asian Americans as one of the race/ethnicity groups (*n* = 13). Only a few projects examined Asian-American ethnic groups (*n* = 8), country of origin (*n* = 3), foreign-born status (*n* = 1), or spoken language (*n* = 1). It is unclear whether currently active studies will examine and report on ethnic differences. The extent of reporting on ethnic differences may depend on the availability of a sufficient sample size within each group.

**Figure 1.**
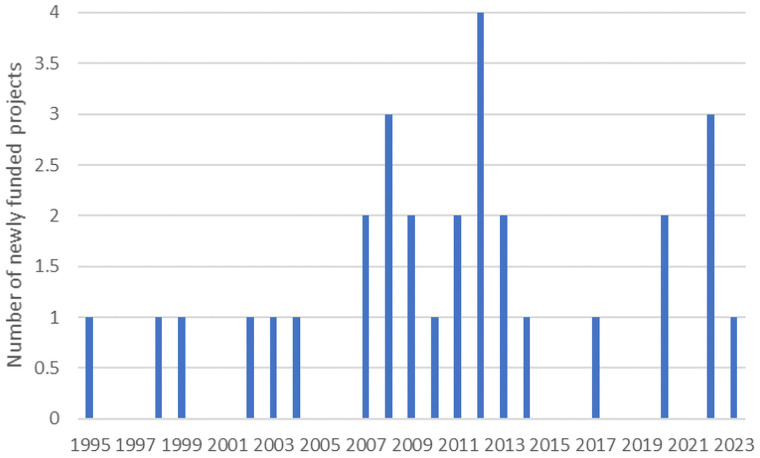
NIH-funded projects focused on Asian Americans and substance use disorder. Abbreviation: NIH, National Institutes of Health.

Figure 1 illustrates the trends in NIH-funded projects from 1995 to 2023, revealing a scarcity of funded projects aimed at studying SUDs among Asian Americans. Out of the 30 funded projects, the majority focused on any substance use (*n* = 17), followed by alcohol only (*n* = 10), club drugs (*n* = 1), and cocaine and alcohol (*n* = 1). Most of the projects aimed to understand the epidemiology and behavioral risk factors for using substances or accessing treatment services, and a significant portion focused on special populations such as college students (*n* = 3), adolescents/youth or young adults (*n* = 7), gay/bi/men who have sex with men (*n* = 1), and elderly populations (*n* = 1). Most of the research was funded by the National Institute on Drug Abuse (*n* = 12), followed by the National Institute on Alcohol Abuse and Alcoholism (*n* = 11), NIMHD (*n* = 5), and the National Institute of Mental Health (*n* = 2). Additionally, less than one-third of the projects were funded by the R01 mechanism (*n* = 8), of which none were intervention studies. There was 1 R24 grant focused on piloting an intervention using a community participatory research design.

Few projects on SUD in Asian Americans have been funded by the NIH, reflecting the pervasiveness of the “model minority stereotype,” which suggests that Asian Americans are a healthy and monolithic community.^25^

One of the primary limitations of this analysis is its reliance on data extracted from the NIH RePORTER database using the search terms. While we made every effort to extract relevant information accurately, the database may not provide exhaustive details on the specific factors considered within each grant. This analysis is constrained by the available data and may not capture the full scope of research priorities and focus areas related to SUD in Asian-American communities.

Advocacy efforts have focused on improving the quality of data for Asian-American ethnic groups in addiction health services research.^28^ Currently, there is a significant gap in research, and there are no publicly available national-level data on addiction health services research by Asian-American ethnic groups. The NSDUH and the Treatment Episode Data Set (TEDS), 2 major sources of national-level data on substance use, do not publish Asian-American ethnic group data. Although NSDUH has Asian-American ethnic group data available through the Restricted Data Center, TEDS does not include Asian-American ethnic group data. The NSDUH primarily offers interviews in English and Spanish.^29^ NSDUH interviewers may use language-assistance tools to facilitate interviews in other languages when necessary. However, given the linguistic diversity within the Asian-American population, the lack of language options beyond English can hinder the participation and accurate representation of individuals who are not proficient in English.^30^ The exclusion of non–English-speaking Asian Americans or individuals with LEP from the survey may limit our understanding of substance use and mental health patterns within this population. Both SUD and LEP populations are more likely to be of low socioeconomic status (SES).^12,31^ Consequently, underrepresenting individuals of low SES may impact the accuracy of SUD prevalence estimates. It is imperative to understand the relationship between LEP and low SES in Asian-American communities to fully assess the implications of this exclusion. Additionally, due to the limited sample size of Asian Americans in these datasets, there are challenges in obtaining a sufficiently large sample within a single year. Many studies have been pooling data across multiple years to overcome the limitations posed by the small sample size in order to derive more reliable conclusions. Therefore, the implication of pooling data has led to collapsing subgroups into broad categories, hindering understanding of the prevalence of SUD and appropriate treatment strategies for Asian-American communities. Where possible, there is a need to harness existing available data and use innovative data strategies to support disaggregated data analysis.^3^

To adequately diagnose current problems and address future concerns among this understudied population, there is an urgent need to skillfully harness the data available with a focus on ethnic group variations. By examining these variations, we can gain insights into the unique challenges and needs of different ethnic groups. For example, alcohol use may be more prevalent among younger Korean-American adults compared with younger Asian-Indian adults, indicating the influence of cultural and contextual factors specific to each group.^32^ Additionally, immigration is protective against alcohol use among Chinese Americans, Filipino Americans, and Asian-Indian Americans, but not among Korean or Japanese Americans, possibly reflecting unique acculturation experiences and cultural norms within ethnicity.^33^ By utilizing an intersectionality approach and examining variations of SUD by age, ethnicity, and gender, we can gain a more nuanced understanding of substance use trends and tailor interventions that address the specific needs and challenges faced by different subgroups within the Asian-American population.^34,35^

However, it is important to acknowledge the challenges in addressing these differences and making informed decisions within Asian-American ethnic groups. The lack of quality Asian-American ethnic group race/ethnicity data, including individuals with LEP, in addiction health services research presents a significant challenge in addressing addiction disparities that exist among Asian-American ethnic groups. Approximately one-third of the Asian-American population have LEP, and neglecting to offer surveys in Asian languages denies them the opportunity to contribute their valuable perspectives and experiences.^32^

Recognizing and addressing the cultural and contextual factors that influence the experiences of Asian Americans in SUD treatment is crucial for achieving equitable and effective care.^36^ Through community-based and patient-centered research and the implementation of culturally and linguistically tailored interventions, we can make substantial progress in improving treatment access, utilization, and outcomes for Asian Americans seeking SUD treatment.

Despite recent attention given to the lack of health data among Asian-American communities during the COVID-19 pandemic, this population remains understudied.^37^ It is important to include Asian Americans and examine ethnic group differences in efforts to understand health disparities related to substance use and access to SUD treatment services.

## Broad themes and implications for other understudied populations

Historically, Asian Americans have been understudied in national health research, especially in the context of SUD research. This commentary underscores the urgent need for targeted SUD support, research initiatives, resources, and programs that specifically cater to the diverse Asian-American population, both as a collective and when disaggregated by ethnic groups. The insights gained from examining the Asian-American experience with SUD can offer valuable insights for research involving other understudied populations. There is tremendous heterogeneity across ethnic groups, such as Native Hawaiians, Pacific Islanders, Caribbean Black, Latinx, and Middle Eastern and North African (MENA) communities. These groups encounter similar challenges, such as issues related to data disaggregation, language barriers, and the burden of negative stereotypes. Native Hawaiians and Pacific Islanders, for instance, are diverse not only in terms of their historical experiences but also in their geographic regions, cultural and linguistic identities, and citizenship and immigration statuses. Although they have often been grouped with Asian-American communities, there is a growing recognition of the need to distinguish them as unique populations. Similarly, the Latinx population comprises individuals from Central and South American backgrounds, who exhibit vast diversity in terms of culture, language, migration, and immigration experiences. Within the MENA population, there exists an emerging racial and ethnic group that has been largely overlooked in data-collection efforts. Much like the challenges faced by Asian-American populations in classifying ethnic groups, the MENA community's recognition and classification within US racial and ethnic categories remain subjects of ongoing debate and ambiguity. The absence of data disaggregation or their inclusion within the White category in health disparity statistics obscures the distinct health and social patterns within the MENA community.^38^ Disaggregating data by specific ethnic groups within these populations can unveil crucial insights into SUD prevalence, treatment needs, and completion rates. Exploring the nuances within racial and ethnic groups, considering factors such as age and migration experience, is an indispensable and encouraging pursuit. Enhancing access to treatment and support for all communities necessitates addressing cultural norms and values, promoting cultural competence in health care services, and improving the availability of linguistically appropriate services, education, and programs. To achieve equitable access to SUD treatment, it is imperative to allocate resources effectively, develop culturally sensitive interventions, and tackle systemic barriers, including limited linguistic accessibility. These efforts are essential to ensure that individuals from diverse backgrounds can readily access the support they need to effectively address SUD and ultimately achieve improved health outcomes.

## Contribution statement

All authors were involved in drafting and critically revising the manuscript for important intellectual content.

## Supplementary Material

qxad058_Supplementary_Data
